# Arabic Validation of the Problematic Pornography Consumption Scale: Psychometric Properties, Measurement Invariance, and Gender-Specific Network Analysis

**DOI:** 10.1007/s10508-026-03532-z

**Published:** 2026-10-14

**Authors:** Abdulrhman Helal, Mohamed Mahmoud Marey, Esraa Reda Abdelaziz, Ahmed Hassan, Mahmoud M. Elhady, Yomna Ahmed Elgohry, Elaf Ahmed Basha, Samantha N. Sallie

**Affiliations:** 1 Faculty of Medicine, Benha University, Benha, Qalubiya 13518 Egypt; 2 Benha Students’ Innovation and Research Association, Benha, Qalubiya Egypt; 3 Faculty of Medicine, Alexandria University, Alexandria, Egypt; 4 Medical Research Group of Egypt, Negida Academy, Arlington, MA USA; 5 Faculty of Medicine, Luxor University, Luxor, Egypt; 6 Faculty of Medicine, Al-Azhar University, Cairo, Nasr City, Egypt; 7 Faculty of Medicine and Health Sciences, Omdorman Islamic University, Khartoum, Sudan; 8 Department of Psychiatry, University of Cambridge, Cambridge, UK

**Keywords:** Problematic pornography use, Problematic Pornography Consumption Scale, Gender differences, Arabic-speaking populations, DSM-5

## Abstract

Problematic pornography use (PPU) remains understudied in Arabic-speaking populations, partly due to the limited availability of culturally validated assessment tools. This study translated and validated the Arabic version of the Problematic Pornography Consumption Scale (PPCS) and examined gender-based differences in symptom structure. A total of 959 Egyptian university students who watched pornography at least once per week (65.1% male, 34.9% female) completed the Arabic PPCS, along with standardized measures of depression, anxiety, perceived stress, and problematic internet use. Confirmatory factor analysis supported the original six-factor model (CFI = 0.950, RMSEA = 0.075, SRMR = 0.031), with the scale demonstrating excellent internal consistency (Cronbach’s *α* = .95) and moderate-to-strong construct validity as indicated by positive correlations with measures of depression (*r* = .42), anxiety (*r* = .37), perceived stress (*r* = .37), and problematic internet use (*r* = .46; all *p* < .001). Overall, 22% of participants were identified as at risk for PPU. Measurement invariance across gender indicated the scale functioned similarly for males and females. Network analysis further revealed gender-specific symptom patterns, with tolerance emerging as the most central symptom among males and withdrawal most central among females. These findings provide initial validation of the Arabic PPCS and highlight the importance of culturally informed and gender-sensitive approaches to understanding PPU in Arabic-speaking populations.

**Supplementary Information:**

The online version contains supplementary material available at https://doi.org/10.1007/s10508-026-03532-z.

## Introduction

Access to online pornography has increased substantially over the past two decades (Lewczuk et al., 2022). Although pornography use is common, a subset of individuals experiences patterns characterized by impaired control, continued engagement despite negative consequences, and associated distress or functional impairment, often referred to as problematic pornography use (PPU) (Bőthe et al., 2020a, 2020b; Marshall & Miller, 2019). Evidence distinguishing individuals with non-problematic high-frequency use further supports the view that behavioral dysregulation and functional impairment, rather than frequency alone, are central to identifying clinically relevant patterns of problematic use (Bőthe et al., 2020a, 2020b).

In much of the empirical literature, PPU is examined within behavioral addiction frameworks grounded in components such as salience (preoccupation with pornography), mood modification (using pornography to regulate emotions), tolerance (needing more intense use to achieve the same effect), withdrawal (negative feelings during abstinence), conflict (interpersonal or intrapsychic problems from use), and relapse (unsuccessful attempts to reduce use) (Griffiths, 2005; Vieira & Griffiths, 2024). However, the broader conceptualization of PPU remains contested. This lack of consensus is reflected in classification systems: Compulsive Sexual Behavior Disorder (CSBD), which may include PPU, is classified in ICD-11 as an impulse control disorder rather than a behavioral addiction and was not included in DSM-5 due to insufficient evidence (Grubbs et al., 2020; World Health Organization, 2019).

Critically, concerns have been raised regarding the validity of applying component-based addiction models to problematic sexual behavior, particularly given limited and inconsistent evidence for core features such as tolerance and withdrawal (Castro-Calvo et al., 2022; Sassover et al., 2022). More broadly, reliance on symptom-based frameworks has been criticized for failing to adequately capture the psychological and learning processes underlying the development and maintenance of these behaviors (Castro-Calvo et al., 2022). In response, process-based accounts have been proposed that emphasize mechanisms implicated in recognized substance and behavioral addictions, including shifts from goal-directed to stimulus-driven behavioral control, the increasing dominance of compulsivity over impulsive reward-seeking, and the role of relative outcome utility in shaping whether sexual behavior becomes a primary strategy for emotional regulation (Castro-Calvo et al., 2022; Dalley & Roiser, 2012; Everitt & Robbins, 2016; Perales et al., 2020; Robinson & Berridge, 2025). Other perspectives foreground the role of moral incongruence, suggesting that distress associated with pornography use may reflect conflict between sexual behavior and personal or religious values rather than compulsive or dysregulated use per se (Vaillancourt-Morel & Bergeron, 2019). Taken together, these perspectives highlight the conceptual complexity surrounding PPU and underscore the importance of reliable approaches to its assessment.

One widely used instrument for assessing PPU is the Problematic Pornography Consumption Scale (PPCS), a self-report measure developed within Griffiths’ (2005) behavioral addiction framework that demonstrates a stable six-factor structure across diverse samples (Bőthe et al., 2018; Fernandez & Griffiths, 2021). PPCS scores and the other related measures of PPU have been associated with a range of psychological and behavioral factors implicated in compulsive sexual behavior, including internalizing symptoms such as depression, anxiety, and perceived stress (Kraus et al., 2016), behavioral dysregulation such as hypersexuality and problematic gaming (Koós et al., 2022; Mestre-Bach et al., 2024), and substance use (Dubois et al., 2025). The scale has been translated and validated across multiple languages, including Chinese, Persian, Serbian, and Spanish (Bőthe et al., 2024; Villena-Moya et al., 2024), and cross-cultural comparisons have demonstrated substantial variation in PPU occurrence rate estimates across languages and countries, highlighting the influence of sociocultural context on pornography use and reporting (Bőthe et al., 2024). However, an Arabic version of the PPCS has not yet been formally validated, leaving a gap in validated tools for assessing PPU in Arabic-speaking populations (Eljawad et al., 2021; El-Kak, 2013), a limitation identified as a critical barrier to research in the region (Malaeb et al., 2023).

In a large cross-national survey of Arab populations, pornography use was widely reported, with 50% of participants indicating use within the past week (Eljawad et al., 2021), suggesting that engagement with pornographic content occurs despite prevailing cultural and legal restrictions in many parts of the region. This gap is particularly relevant in countries such as Egypt, where pornography is both legally restricted and socially discouraged. Egyptian law prohibits the production, distribution, and possession of pornographic material, with violations carrying penalties including substantial fines of up to EGP 2 million and imprisonment of 1–5 years (AlAshry, 2022; Shishkina & Issaev, 2018; Zagloul et al., 2022). Beyond these legal restrictions, pornography consumption is widely regarded as socially taboo within conservative Islamic cultural contexts, where such behaviors are often framed as morally prohibited (Eljawad et al., 2021).

Despite these restrictions, internet access among young adults in Egypt is widespread, with nearly 90% of young adults reporting daily internet use (Central Agency for Public Mobilization and Statistics [CAPMAS], 2021), increasing the likelihood of exposure to sexually explicit material. In addition, large-scale survey research suggests that many individuals access restricted online content through virtual private networks (VPNs), with estimates indicating that between 50 and 70% of youth use such tools to circumvent content restrictions (AlAshry, 2022; Shishkina & Issaev, 2018). These factors suggest that pornography consumption persists despite legal and sociocultural constraints, highlighting the need for culturally appropriate tools for studying PPU in the region.

Within sociocultural contexts where pornography use is strongly stigmatized, gender norms may shape how such behaviors are experienced and reported. Across many studies, males consistently report higher levels of pornography consumption and higher occurrence of PPU symptoms than females (Bőthe et al., 2024; Fernandez & Griffiths, 2021). However, these differences may partly reflect sociocultural influences on reporting behavior. For example, in contexts where pornography use is particularly stigmatized for women, females may report greater distress associated with pornography consumption even at lower levels of use (Ince et al., 2025). Establishing measurement invariance is therefore necessary to determine whether observed gender differences in PPU reflect true differences in the underlying construct rather than differences in how individuals interpret or respond to scale items (Putnick & Bornstein, 2016).

Beyond establishing the psychometric properties of the scale, exploring the structure of PPU symptoms may provide further insight into how PPU manifests within specific populations. Network analysis conceptualizes psychopathology as arising from dynamic interactions among symptoms rather than from a single underlying latent construct (Borsboom, 2017). This approach has been applied to a range of psychological and behavioral conditions, including alcohol use disorders (Anker et al., 2017), anxiety (Beard et al., 2016), depression (Schweren et al., 2018), and hypersexuality (Werner et al., 2018). Recent work has also applied network models to PPU, suggesting that the relative importance of specific symptoms may vary across cultural contexts (Chen et al., 2021). However, the symptom network structure of PPU remains unexplored in Arabic-speaking populations. Applying this approach to an Egyptian sample provides an important first step in establishing a baseline symptom structure within this cultural context, while also allowing exploration of potential gender differences in symptom organization.

The present study aimed to translate, culturally adapt, and validate the Arabic version of the PPCS among Egyptian university students. We evaluated the psychometric properties of the PPCS, including internal consistency, concurrent validity, and factor structure. We tested measurement invariance across gender to determine whether the scale operates equivalently for male and female participants.

In addition, we applied network analysis to explore the interrelationships among PPU symptoms within this population and to examine potential gender differences in symptom centrality. Based on the previous research, we hypothesized that the Arabic PPCS would demonstrate strong internal consistency and a six-factor structure consistent with Griffiths’ (2005) behavioral addiction model. We further expected that the structure of symptom networks would differ across genders, reflecting potential variation in how PPU manifests among male and female students.

## Method

### Participants

Participants were Egyptian university students recruited through online distribution of the survey. Eligibility criteria included regular internet access and pornography engagement at least once per week. This frequency threshold was used to distinguish consistent engagement from occasional exposure and to ensure that the PPCS assessed relatively stable behavioral patterns rather than sporadic use. Restricting the sample to regular users also helped reduce potential misclassification of distress driven primarily by moral or religious conflict rather than sustained behavioral involvement, which has been observed among individuals reporting infrequent pornography use (Mestre-Bach et al., 2021). Pornography use frequency was assessed using a screening question administered at the beginning of the survey.

Participants were excluded if they reported a history of diagnosed psychiatric or neurological disorders, assessed via a self-report screening item (“Have you ever been diagnosed with a psychiatric disorder by a qualified healthcare professional?” Yes/No). This criterion was applied to reduce potential confounding effects of pre-existing psychopathology on analyses examining associations between PPCS scores and psychological distress measures (depression, anxiety, and stress).

Responses were also excluded if survey completion time was less than nine minutes, based on pilot testing, indicating that shorter durations were unlikely to reflect attentive responding (see Supplementary Material, Section 1 (S1)). Incomplete submissions were removed before analysis. A total of 3,135 university students were initially screened. Of these, 2039 were excluded for reporting pornography use less than once per week. Additional exclusions included self-reported psychiatric diagnoses (*n* = 29), survey completion times below the attentiveness threshold (*n* = 30), and incomplete responses (*n* = 78), resulting in a final analytical sample of *N* = 959 participants.

The required sample size was determined based on methodological recommendations for confirmatory factor analysis (CFA; *N* = 10–20 per item) (Kyriazos, 2018), measurement invariance testing (*N* ≥ 200 per group) (Meade & Bauer, 2007), and network analysis (*N* ≥ 250–300 per subgroup) (Epskamp et al., 2018a). Given the PPCS-18 item structure, the minimum requirements were 360 for CFA, 400 for invariance testing, and 500–600 for network analysis. The final sample of 959, recruited through convenience and snowball sampling, exceeded all thresholds.

### Procedure

PPCS was translated into Arabic following the forward–backward translation procedure recommended by the International Test Commission guidelines (Bartram et al., 2018). Two independent bilingual translators produced separate Arabic translations, which were synthesized into a single version. A third independent translator, blinded to the original English scale, conducted back-translation. An expert committee comprising specialists in psychology, psychiatry, and psychometrics reviewed all versions to resolve discrepancies and ensure conceptual and cultural equivalence. Full details of the translation process can be found in the Supplementary Material, Section 1, Part 2.

A pilot study with 25 university students (April 5–10, 2024) evaluated the clarity, cultural relevance, and comprehensibility of the Arabic PPCS-18. Feedback from the pilot informed minor refinements to wording and structure. Details regarding completion time estimates and specific adjustments are provided in Supplementary Material, Section 1.

The main cross-sectional study was conducted from April 17 to May 28, 2024, using a self-administered online survey hosted on Google Forms. Electronic informed consent was obtained before data collection. The online format was used to enhance accessibility and anonymity and to reduce social desirability bias associated with sensitive topics (Krumpal, 2013).

### Measures

#### Problematic Pornography Consumption Scale

PPU was assessed using the PPCS, an 18-item self-report instrument grounded in Griffiths’ (2005) six-component model of behavioral addiction. The scale evaluates six dimensions: salience, mood modification, conflict, tolerance, relapse, and withdrawal. Items were rated on a 7-point Likert scale ranging from 1 (*never*) to 7 (*always*). Total scores range from 18 to 126, with higher scores indicating greater levels of PPU. A total score of 76 or higher has been proposed as indicative of possible problematic use (Bőthe et al., 2018).

#### Patient Health Questionnaire-9

Depressive symptoms were measured using the Arabic version of the 9-item Patient Health Questionnaire (PHQ-9). Items were rated on a 4-point scale from 0 (*not at all*) to 3 (*nearly every day*), with total scores ranging from 0 to 27. Higher scores indicate greater depressive symptom severity. Severity categories are defined as minimal (0–4), mild (5–9), moderate (10–14), moderately severe (15–19), and severe depression (20–27) (Kroenke et al., 2001; Sawaya et al., 2016).

#### Generalized Anxiety Disorder-7

Anxiety symptoms were assessed using the Arabic version of the 7-item Generalized Anxiety Disorder scale (GAD-7). The 7 items were rated on a 4-point Likert scale ranging from 0 (*not at all*) to 3 (*nearly every day*), with total scores ranging from 0 to 21. Severity categories include minimal (0–4), mild (5–9), moderate (10–14), and severe anxiety (15–21) (Sawaya et al., 2016; Spitzer et al., 2006).

#### Perceived Stress Scale-10

Perceived psychological stress was measured using the Arabic version of the 10-item Perceived Stress Scale (PSS-10). Items were rated on a 5-point Likert scale ranging from 0 (*never*) to 4 (*very often*), with total scores ranging from 0 to 40. Scores are categorized as low stress (0–13), moderate stress (14–26), and high stress (27–40) (Chaaya et al., 2010).

#### Internet Addiction Test-20

Problematic Internet use was assessed using the Arabic version of the 20-item Internet Addiction Test (IAT-20). Each item was rated on a 5-point Likert scale ranging from 1 (*rarely*) to 5 (*always*). Total scores range from 20 to 100, with severity classifications defined as normal (0–30), mild (31–49), moderate (50–79), and severe (80–100) problematic internet use (Hawi, 2013).

#### Social Desirability Assessment

Social desirability bias was assessed using two culturally adapted items derived from the Marlowe–Crowne Social Desirability Scale (MC-SDS). The items assessed tendencies toward socially desirable responding and external approval seeking. Participants rated the following statements on a 5-point Likert scale:

1. “When I don’t know something, I don’t mind admitting it.”
2. “I always try to achieve the highest academic grades to gain appreciation from people around me.”

Response options ranged from 1 (*strongly disagree*) to 5 (*strongly agree*), with the second item reverse-scored. The academic achievement item was culturally adapted to reflect social approval motives relevant to Egyptian university students. Scores of 8 or higher were classified as indicating elevated socially desirable responding based on a median split approach commonly used when population norms are unavailable (Sousa & Rojjanasrirat, 2011).

#### Sociodemographic Variables

Participants provided self-reported sociodemographic information, including age, gender, marital status, nationality, region of residence, urban versus rural residence, field of study (health-related vs. non-health-related), academic year, family history of psychiatric illness, and presence of chronic medical conditions. These variables were included for descriptive purposes and to examine group differences in PPU risk.

### Statistical Analysis

All analyses were conducted using IBM SPSS Statistics version 26 and JASP version 0.18.3. Descriptive statistics summarized demographic characteristics and scale scores. Chi-square tests examined associations between categorical variables and PPU risk status. Effect sizes were calculated using Cramér’s V, with values of.10,.30, and.50 interpreted as small, medium, and large effects, respectively (Ialongo, 2016). Social desirability was analyzed as a categorical variable (socially desirable vs. non-socially desirable responding) to examine whether response bias influenced the likelihood of reporting PPU risk.

Internal consistency of the Arabic PPCS was evaluated using Cronbach’s *α*, item–total correlations, and inter-item correlations. CFA was conducted to test the six-factor structure of the PPCS-18 using the Diagonally Weighted Least Squares (DWLS) estimator, which is appropriate for ordinal Likert-type data. The six-factor model was specified a priori based on prior validations of the instrument (Bőthe et al., 2024). Given this established structure, CFA was used to directly test factorial validity while avoiding overlaps between exploratory and confirmatory procedures within the same sample (DiStefano & Hess, 2005). Sampling adequacy of the correlation matrix was assessed using the Kaiser–Meyer–Olkin (KMO) measure (> 0.90 indicating excellent suitability) and Bartlett’s sphericity test (*p* < 0.001). Model fit was evaluated using the Comparative Fit Index (CFI) and Tucker–Lewis Index (TLI) (≥ 0.95 indicating good fit), Normed Fit Index (NFI) and Parsimony Normed Fit Index (PNFI), the Root Mean Square Error of Approximation (RMSEA) (≤ 0.06 good, ≤ 0.08 acceptable), and the Standardized Root Mean Square Residual (SRMR) (≤ 0.08 acceptable) (Xia & Yang, 2019).

Measurement invariance across gender was assessed through multi-group CFA with the DWLS estimator. Invariance testing proceeded hierarchically: configural (same factor structure), metric (equal factor loadings), and scalar (equal item intercepts) models. Model fit was evaluated using multiple indices: Chi-square statistic (*χ*^2^), CFI (≥ 0.95 indicates good fit), TLI (≥ 0.95 indicates good fit), RMSEA with 90% confidence intervals (≤ 0.08 acceptable, ≤ 0.06 good), and SRMR. Measurement invariance was supported if changes in fit indices between increasingly constrained models met recommended thresholds (ΔCFI <.01 and ΔRMSEA <.015) (D’Urso et al., 2022; Xia & Yang, 2019).

Construct validity was assessed using Spearman partial correlations between PPCS-18 and related psychological measures (PHQ-9, GAD-7, PSS-10, and IAT-20). Analyses controlled for social desirability as well as demographic and psychosocial factors that may influence pornography-related behaviors or reporting patterns, including sex, study field, housing area (rural vs. urban), family psychiatric history, sleep duration, and chronic illness. These variables were coded as follows for the partial correlations: sex (0 = female, 1 = male), study field (0 = non-health-related, 1 = health-related), housing area (0 = rural, 1 = urban), family psychiatric history (0 = no, 1 = yes), and chronic illness (0 = no, 1 = yes). Social desirability scores and sleep hours were entered as continuous variables. These variables were included to account for potential reporting bias, differences in exposure and social context, and individual vulnerability factors previously associated with PPU and related psychological outcomes (Harper & Hodgins, 2016; Pham et al., 2025; Seyedzadeh Dalooyi et al., 2023). False discovery rate correction was applied to adjust for multiple comparisons. All inferential statistical tests were two-tailed with a significance level set at *p* <.05.

Symptom networks were estimated separately by gender using the Extended Bayesian Information Criterion Graphical Least Absolute Shrinkage and Selection Operator (EBICglasso) with hyperparameter *γ* = 0.5, a regularization approach that identifies robust connections while limiting overfitting (Foygel & Drton, 2010). Networks were visualized using the Fruchterman–Reingold algorithm with fixed coordinates to facilitate gender comparisons. Node influence was quantified using four centrality indices: betweenness (frequency on shortest paths), closeness (inverse average shortest path length), strength (sum of absolute edge weights), and expected influence (sum of signed weights). Expected influence reflects the summed signed edge weights connected to a node and is particularly informative in psychological networks where relationships between variables may vary in direction and magnitude (Bendahman & Lotfi, 2024; Epskamp et al., 2018a, 2018b). All indices were *z*-standardized to facilitate comparison. Network stability was evaluated via nonparametric bootstrapping (1000 resamples). Edge weight confidence intervals (CIs) assessed precision, with intervals excluding zero indicating robust associations. Centrality stability was examined using the case-dropping bootstrap, yielding a correlation-stability coefficient; with values > 0.25 indicating acceptable, and > 0.50 indicating good stability (van Borkulo et al., 2023). Centrality-difference plots (*α* =.05) were used to assess whether centrality estimates differed significantly (significant contrasts shown in black; non-significant in gray; node-strength values reported in white boxes). Due to unequal gender distribution (≈ 2:1 male: female), a formal network comparison test was not conducted, as simulation studies show limited power to detect subtle structural differences under substantial group imbalance. Instead, standardized centrality profiles were compared descriptively across gender-specific networks (Epskamp et al., 2018a; van Borkulo et al., 2023).

To assess robustness, a random subsample of 494 participants was drawn, and the primary analyses were repeated. Results were largely consistent and are presented in Supplementary Materials File, Section 3.

## Results

### Sample Characteristics

The final analyzed sample consisted of 959 participants. The mean age of participants was 21 years (SD = 2.26; range = 18–38), with 65.1% identifying as male (*N* = 624) and 34.9% as female (*N* = 335). The majority were single (98.5%, *N* = 945). PPU risk was identified in 21.8% of the sample (*N* = 209), with significantly higher rates among males (25.5%) compared to females (14.6%) (*χ*^2^ = 15.119, *p* <.001) and those who responded in a less socially desirable manner (24.2% vs. 14.8%) (*χ*^2^ = 9.62, *p* = 0.002). Additional sample characteristics are presented in Table 1.

**Table 1:** Sample characteristics and association with problematic pornography use risk

Variable	Non-problematic pornography use (n, %)	Problematic pornography use risk (n, %)	Total (n, %)	*p*-value (Cramér’s V effect size for significant variables)
*Anxiety*				
No anxiety	192 (25.5%)	14 (6.7%)	206 (21.5%)	< 0.001* **(0.26)**
Mild anxiety	223 (29.8%)	49 (23.6%)	272 (28.4%)	
Moderate anxiety	182 (24.2%)	48 (23.1%)	230 (24%)	
Severe anxiety	154 (20.5%)	97 (46.6%)	251 (26.1%)	
*Depression*				
No depression	103 (93.6%)	7 (6.4%)	110 (11.5%)	< 0.001* **(0.30)**
Mild depression	206 (90.0%)	23 (10.0%)	229 (24%)	
Moderate depression	185 (80.1%)	46 (19.9%)	231 (24.1%)	
Moderate–severe depression	132 (73.3%)	48 (26.7%)	180 (18.8%)	
Severe depression	125 (59.8%)	84 (40.2%)	209 (21.8%)	
*Stress*				
Mild stress	110 (92.4%)	9 (7.6%)	119 (12.4%)	< 0.001* **(0.27)**
Moderate stress	539 (80.8%)	128 (19.2%)	667 (69.6%)	
Severe stress	102 (59.0%)	71 (41.0%)	173 (18%)	
*Sex*				
Male	465 (74.5%)	159 (25.5%)	624 (65.1%)	< 0.001* **(0.12)**
Female	286 (85.4%)	49 (14.6%)	335 (34.9%)	
*Marital Status*				
Single	741 (78.4%)	204 (21.6%)	945 (98.5%)	0.328
Married	7 (63.6%)	4 (36.4%)	11 (1.1%)	
Divorced	3 (100.0%)	0 (0.0%)	3 (0.3%)	
*Nationality*				
Egyptian	696 (78.2%)	194 (21.8%)	890 (92.8%)	0.888
Non-Egyptian	55 (79.7%)	14 (20.3%)	69 (7.2%)	
*Region of Living*				
Greater Cairo and the Delta Region	358 (78.2%)	100 (21.8%)	458 (47.8%)	0.755
ALEX Region	185 (77.1%)	55 (22.9%)	240 (25.0%)	
Canal Region	51 (76.1%)	16 (23.9%)	67 (7.0%)	
Upper Egypt	157 (80.9%)	37 (19.1%)	194 (20.2%)	
*Area of Housing*				
Rural	311 (77.8%)	89 (22.3%)	400 (41.7%)	0.782
City	440 (78.7%)	119 (21.3%)	559 (58.3%)	
*Field of Study*				
Health Related	446 (77.0%)	133 (23.0%)	579 (60.4%)	0.268
Non-Health Related	305 (80.3%)	75 (19.7%)	380 (39.6%)	
*Year of School*				
First	162 (77.1%)	48 (22.9%)	210 (21.9%)	0.552
Second	197 (82.1%)	43 (17.9%)	240 (25.0%)	
Third	147 (78.2%)	41 (21.8%)	188 (19.6%)	
Fourth	113 (73.9%)	40 (26.1%)	153 (16.0%)	
Fifth	67 (78.8%)	18 (21.2%)	85 (8.9%)	
Sixth	65 (78.3%)	18 (21.7%)	83 (8.7%)	
*History of Family Psychiatric Illness*				
Yes	119 (74.8%)	40 (25.2%)	159 (16.6%)	0.291
No	632 (79.0%)	168 (21.0%)	800 (83.4%)	
*Chronic Illness*				
Yes	74 (75.5%)	24 (24.5%)	98 (10.2%)	0.562
No	677 (78.6%)	184 (21.4%)	861 (89.8%)	
*Q1 of social desirability. Academic Motivation: I always strive to achieve the highest academic grades so that I gain the appreciation of the people around me*				
Strongly disagree	56 (67.5%)	27 (32.5%)	83 (8.7%)	
Disagree	143 (76.5%)	44 (23.5%)	187 (19.5%)	
Neutral	218 (78.1%)	61 (21.9%)	279 (29.1%)	
Agree	179 (80.3%)	44 (19.7%)	223 (23.3%)	
Strongly agree	155 (82.9%)	32 (17.1%)	187 (19.5%)	
*Q2 of social desirability. Honesty: When I don’t know something, I have no objection to admitting it*				
Strongly disagree	10 (71.4%)	4 (28.6%)	14 (1.5%)	
Disagree	30 (69.8%)	13 (30.2%)	43 (4.6%)	
Neutral	69 (67.0%)	34 (33.0%)	103 (10.7%)	
Agree	319 (81.4%)	73 (18.6%)	392 (40.9%)	
Strongly agree	323 (79.4%)	84 (20.6%)	407 (42.2%)	
*Social Desirability Answering*				
Answering in a socially desirable way	219 (85.2%)	38 (14.8%)	257 (26.8%)	0.002* **(0.10)**
Answering in a non-socially desirable way	532 (75.8%)	170 (24.2%)	702 (73.2%)	

Bold value indicates statistical significance (*p* < 0.05)

### Descriptive Statistics

PPCS scores in the sample ranged from 18 to 126 (*M* = 55.11, SD = 27.68). Descriptive statistics for other psychological measures, including depressive symptoms (PHQ-9), anxiety symptoms (GAD-7), perceived stress (PSS-10), and problematic internet use (IAT-20), are presented in Table 2.

**Table 2:** Descriptive statistics of the continuous scores

Variable	Mean (SD)	Median (IQR)	Range (Min–Max)
PPCS scores (Problematic Pornography Use)	55.11 (27.68)	49.00 (34.00–71.00)	18.00–126.00
PHQ-9 (Depression Severity)	13.16 (7.13)	13.00 (8.00–19.00)	0.00–27.00
GAD-7 (Anxiety Severity)	10.17 (6.03)	10.00 (5.00–15.00)	0.00–21.00
PSS-10 (Psychological Stress)	20.58 (6.27)	20.00 (17.00–25.00)	2.00–36.00
IAT-20 (Problematic Internet Use)	52.88 (15.76)	51.00 (41.00–62.00)	20.00–100.00
Social Desirability Scores	7.44 (1.62)	8.00 (6.00–9.00)	2.00–10.00

### Reliability Analysis

The Arabic PPCS demonstrated excellent internal consistency (Cronbach’s *α* =.95). Item-total correlations ranged from .54 (Q3) to .82 (Q10), and inter-item correlations ranged from *r* =.28 to *r* =.82 (all *p* <.001) (Supplementary Materials Table S1). No meaningful improvement in internal consistency was observed following the deletion of any item. Detailed reliability statistics are provided in Table 3.

**Table 3:** Problematic Pornography Consumption Scale reliability

Variables	Item-to-total correlations	Item-to-rest correlations	Cronbach’s *α* if the item is dropped
Q1. I felt that porn is an important part of my life	.648***	0.684***	0.952
Q2. I used porn to restore the tranquility of my feelings	.739***	0.753***	0.951
Q3. I felt porn caused problems in my sexual life	.548***	0.531***	0.954
Q4. I felt that I had to watch more and more porn for satisfaction	.798***	0.802***	0.950
Q5. I unsuccessfully tried to reduce the amount of porn I watch	.709***	0.634***	0.953
Q6. I became stressed when something prevented me from watching porn	.698***	0.757***	0.951
Q7. I thought about how good it would be to watch porn	.646***	0.697***	0.951
Q8. Watching porn got rid of my negative feelings	.746***	0.748***	0.951
Q9. Watching porn prevented me from bringing out the best in me	.723***	0.663***	0.952
Q10. I felt that I needed more and more porn in order to satisfy my needs	.820***	0.825***	0.949
Q11. When I vowed not to watch porn anymore, I could only do it for a short period of time	.757***	0.701***	0.951
Q12. I became agitated when I was unable to watch porn	.758***	0.790***	0.950
Q13. I continually planned when to watch porn	.669***	0.710***	0.951
Q14. I released my tension by watching porn	.640***	0.676***	0.952
Q15. I neglected other leisure activities as a result of watching porn	.743***	0.747***	0.951
Q16. I gradually watched more "extreme" porn, because the porn I watched before was less satisfying	.715***	0.706***	0.951
Q17. I resisted watching porn for only a little while before I relapsed	.753***	0.690***	0.952
Q18. I missed porn greatly when I didn’t watch it for a while	.774***	0.780***	0.950
Overall Cronbach’s *α*			**.955**

Bold value indicate the overall scale reliability (Cronbach’s *α*)

^***^p <.001

### Confirmatory Factor Analysis (CFA)

CFA supported the original six-factor structure of the PPCS (salience, mood modification, conflict, tolerance, relapse, withdrawal). Standardized factor loadings ranged from .58 (Conflict: Q3) to .90 (Mood Modification: Q2) (all *p* <.00; see Supplementary Materials Table S2). Model fit indices indicated acceptable model fit, and sampling adequacy of the correlation matrix was confirmed (KMO = 0.957; Bartlett’s test *p* <.001). Full model fit indices are reported in Table 4.

**Table 4:** Confirmatory factor analysis indices

Fit index	Value
Test for Exact Fit	*χ*^2^ = 760, df = 120, *p* <.001
CFI	0.950
TLI	0.936
NFI	0.975
PNFI	0.782
RMSEA [CI]	0.0745 [0.0695, 0.0797]
SRMR	0.0308
Overall (KMO) Test	0.957
Bartlett’s Test	*χ*^2^ (153) = 15,758.01, *p* <.001

*CFA* Confirmatory Factor Analysis, *CFI* Comparative Fit Index, *TLI* Tucker–Lewis Index, *NFI* Normed Fit Index, *PNFI* Parsimony Normed Fit Index, *RMSEA* Root Mean Square Error of Approximation, *CI* Confidence Interval, *SRMR* Standardized Root Mean Square Residual, *KMO* Kaiser–Meyer–Olkin

Measurement invariance across gender was supported at the configural, metric, and scalar levels. The configural model demonstrated acceptable fit, indicating a similar factor structure across groups. Constraining factor loadings (metric invariance) and item intercepts (scalar invariance) resulted in minimal changes in model fit indices, supporting the equivalence of the measurement model across genders. Table 5 shows all fit indices for the three models.

**Table 5:** Measurement invariance testing across gender

Model	*χ*^2^ (df)	CFI	TLI	RMSEA (90% CI)	SRMR	ΔCFI	ΔRMSEA	Decision
Configural Model	505.115 (240)***	0.990	0.987	0.067 (0.059–0.075)	0.034	–	–	Supported
Metric Invariance	466.583 (252)***	0.992	0.990	0.059 (0.050–0.067)	0.038	+ 0.002	− 0.008	Supported
Scalar Invariance	572.601 (336)***	0.991	0.992	0.054 (0.046–0.061)	0.034	− 0.001	− 0.005	Supported

*χ*^*2*^ Chi-square test, *df* degrees of freedom, *CFI* Comparative Fit Index, *TLI* Tucker–Lewis Index, *RMSEA* Root Mean Square Error of Approximation, *CI* Confidence Interval, *SRMR* Standardized Root Mean Square Residual, Δ change in value

^***^p-value tests the null hypothesis of perfect model fit <.001

^***^p <.001

### Construct Validity

PPCS total scores showed moderate-to-strong positive correlations with anxious (*r* = 0.37) and depressive (*r* = 0.42) symptoms, perceived stress (*r* = 0.37), and problematic internet use (*r* = 0.46) scores (all *p* <.001).

**Fig. 1:**
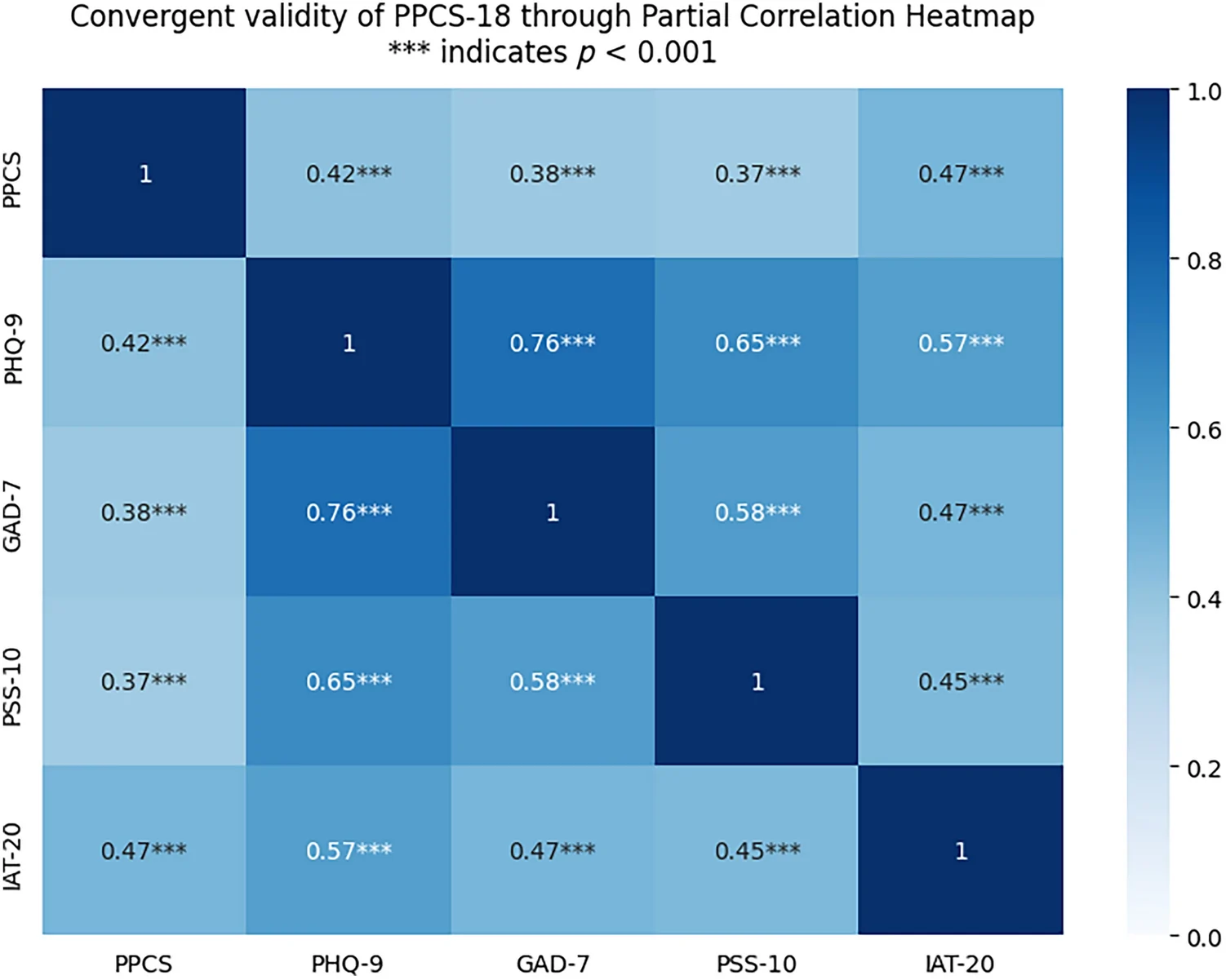
Spearman partial correlation heatmap of the study’s main variables, controlling for social desirability, gender, field of study, area of housing, family history of psychological illness, sleeping hours, and chronic diseases

Crude correlations ranged from *ρ* = 0.37 to 0.49 (all *p* <.001), confirming strong interrelationships. After adjustment, partial ρ values remained robust (0.37–0.45, all *p* <.001), with most correlation coefficients showing minimal shrinkage (range − 8.4% to + 16.4%). No pairs exhibited > 30% reduction or loss of significance, indicating the absence of overcontrol (Lee, 2014). Results are summarized in Supplementary Materials Table S3, and partial correlations are visualized in Fig. 1.

### Network Analysis: Problematic Pornography Consumption Scale Factor Interplay and Gender Differences

#### Overall Network Structure

**Fig. 2:**
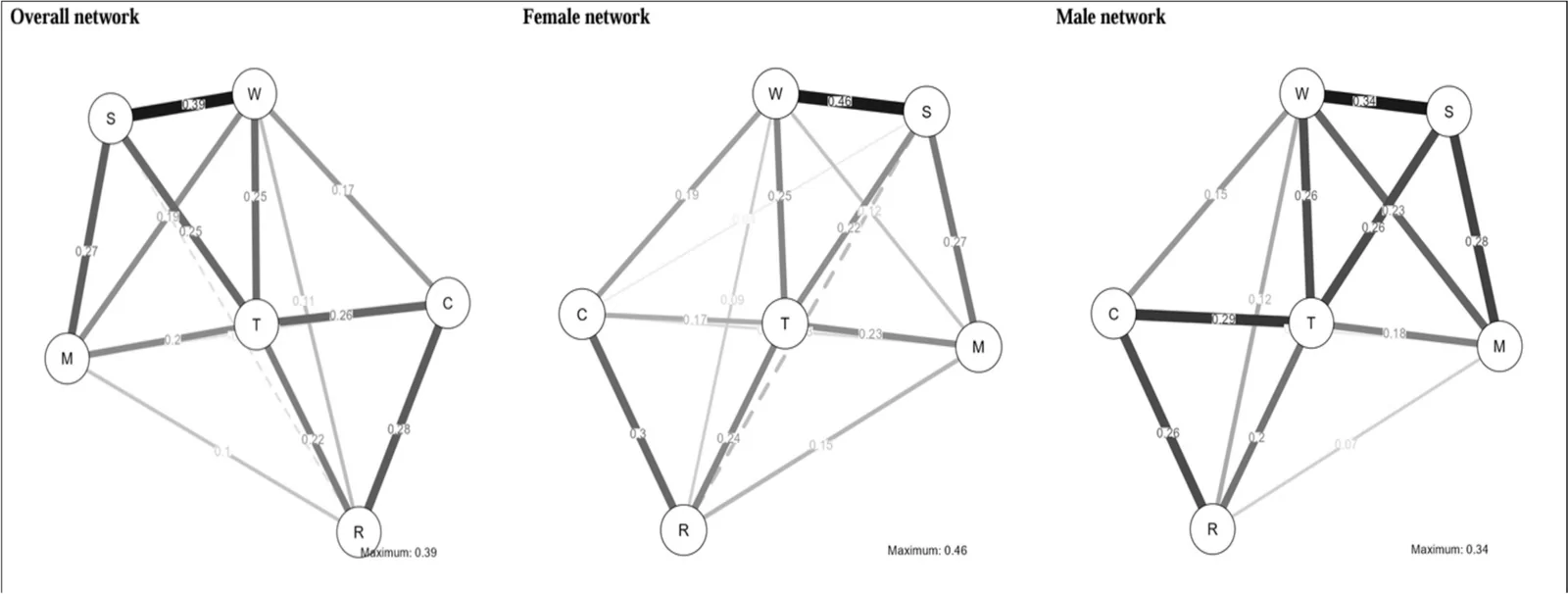
Network structure of PPCS factors for the overall sample, males, and females. Nodes represent the six PPCS factors (salience, mood modification, conflict, tolerance, relapse, withdrawal). Edges (lines) represent the partial correlation coefficients between factors; thicker edges indicate stronger positive associations. The specific edge weights (partial correlation values) are displayed

**Fig. 3:**
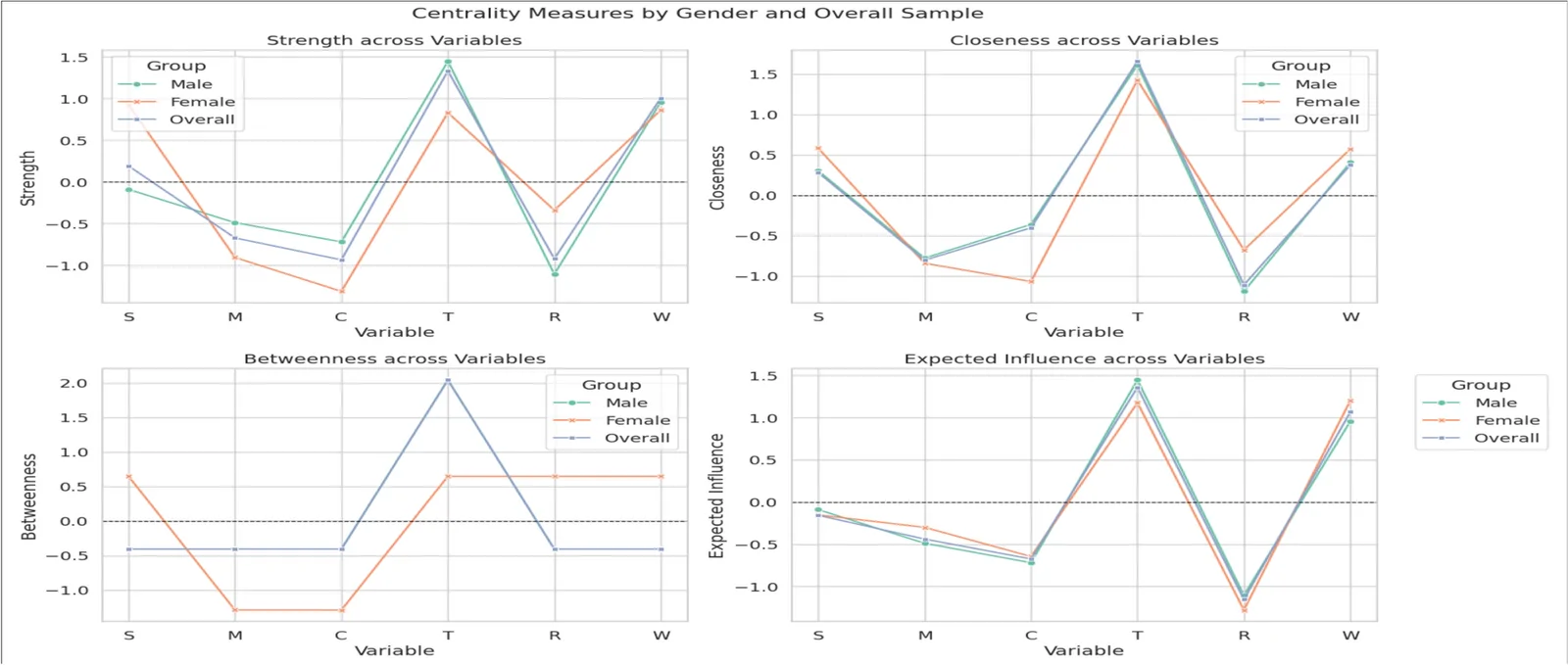
Centrality measures of problematic pornography use factors by gender and overall sample. This figure presents line plots of four distinct centrality measures—Strength, Closeness, Betweenness, and Expected Influence—for each of the six problematic pornography use factors. The factors represented on the x-axis of each subplot are salience (*S*), mood modification (*M*), conflict (*C*), tolerance (*T*), relapse (*R*), and withdrawal (*W*). Values for each centrality measure are displayed on the y-axis, and these values are standardized (z-scores), with a horizontal dashed line indicating zero. Separate lines depict the centrality profiles for Male participants (teal green), Female participants (peach orange), and the Overall sample (slate blue), as indicated in the legend within each subplot. Higher values on the y-axis indicate greater centrality for that specific measure. Notably, in the “Betweenness across Variables” subplot, the centrality values for Male participants and the Overall sample are identical across all factors

Network analysis identified 14 nonzero edges among six PPCS symptoms (sparsity = 0.067). Tolerance emerged as the most central node, showing the highest betweenness (2.04), closeness (1.65), strength centrality (1.33), and expected influence (1.36). The strongest connections were observed between withdrawal and salience (*r* = 0.39), conflict and relapse (*r* = 0.27), and mood modification and salience (*r* = 0.27). No direct edge was observed between conflict and salience. Network structure and centrality indices are illustrated in Figs. 2 and 3.

#### Gender Differences

The male network revealed 13 nonzero edges (sparsity = 0.13), whereas the female network was fully connected with 15 edges (sparsity = 0.000). Centrality patterns differed between groups. In the male network, tolerance emerged as the most central node across betweenness (2.04), closeness (1.61), strength (1.44), and expected influence (1.44), followed by withdrawal. In the female network, withdrawal showed the highest expected influence (1.20), whereas salience showed the highest strength (0.92), and tolerance showed the highest closeness (1.42). Betweenness values were shared across several nodes (tolerance, withdrawal, relapse, and salience; 0.64).

The strongest edge in both networks was observed between withdrawal and salience, with a stronger association in the female network. Some structural differences were also observed. For example, the relapse–salience connection was absent in the male network but was negative in the female network, and the conflict–salience edge appeared only in the female network. Overall network structures and centrality profiles are illustrated in Fig. 3. Network stability and accuracy analyses based on 1000 bootstrap samples are presented in the Supplementary Material, Section 2 part 4.

## Discussion

This study evaluated the psychometric properties of the Arabic version of the PPCS among Egyptian university students who reported pornography use at least once per week. The Arabic PPCS demonstrated excellent internal consistency and replicated the original six-factor structure proposed within Griffiths’ (2005) behavioral addiction framework. Item-level analyses supported the retention of all items, with strong item–total correlations and appropriate factor loadings, indicating that each item contributes meaningfully to the measurement of PPU. These findings support the structural validity of the scale in this Arabic-speaking sample and are consistent with previous cross-cultural validations (Alidost et al., 2022; Bőthe et al., 2024; Chen et al., 2021). The CFA results reinforce the transcultural applicability of the six-component behavioral addiction framework, including in settings with stricter norms surrounding sexual behavior.

Male participants reported higher PPCS scores, consistent with global trends of greater pornography use and addiction-like symptoms among men (Kraus et al., 2016). Measurement invariance across gender was supported at the configural, metric, and scalar levels, indicating that the PPCS measures the construct equivalently in male and female participants. Establishing measurement invariance is particularly important in sociocultural contexts where gender norms surrounding sexuality may influence reporting patterns (El-Kak, 2013). These findings therefore support the use of the Arabic PPCS for meaningful comparisons of PPU symptoms across gender in Arabic-speaking populations.

PPCS scores were positively associated with measures of depression, anxiety, stress, and problematic internet use, a pattern that supports the construct validity of the scale and is consistent with previous research linking PPU to broader psychological distress and behavioral dysregulation (Brand et al., 2016; Engelhardt et al., 2025a, 2025b; Kraus et al., 2020). These relationships align with theoretical accounts, suggesting that negative emotional states may operate both as vulnerability factors and as proximal triggers for engagement in problematic internet use (Brand et al., 2016). Over time, these behaviors may become increasingly maintained through negative reinforcement, a process proposed to involve reduced reward sensitivity and heightened craving or emotional reactivity, thereby promoting continued engagement as a means of alleviating distress or dysphoria (Koob, 2013; Koob & Le Moal, 2001; Koob & Volkow, 2016; Voon et al., 2014). The particularly strong association observed with problematic internet use further suggests that PPU may occur within a broader constellation of problematic internet-use behaviors that share overlapping psychological mechanisms (Brand et al., 2016).

### Occurrence Rate of Problematic Pornography Use in the Egyptian Context

In this present sample of regular pornography users (those reporting use at least once per week), 21.8% met the threshold for PPU risk, comparable to occurrence rates reported in Western samples (15–28%) (Bőthe et al., 2024; Vieira & Griffiths, 2024). Consistent with previous research (Kraus et al., 2016), PPU risk was more common among males (25.5%) than females (14.6%).

Individuals classified as at risk for PPU also reported elevated levels of internalizing symptomatology, with 46.6% reporting severe anxiety symptoms, 40.4% severe depressive symptoms, and 95.6% moderate-to-severe stress. While these findings are consistent with prior work linking PPU to psychological distress (Brand et al., 2016; Engelhardt et al., 2025a, 2025b; Kraus et al., 2020), they should be interpreted with caution given evidence of socially desirable responding in approximately 27% of the sample, which may have contributed to underreporting of stigmatized behaviors.

Interpretation of these findings should consider the conservative sociocultural context of Egypt, where stricter expectations surrounding sexual behavior, particularly for women, may influence how pornography use is experienced and disclosed (El-Kak, 2013). In such contexts, reported distress may reflect not only dysregulated use but also conflict between behavior and personal or cultural values (Grubbs & Perry, 2019).

### Symptom Network Structure and Gender Differences

Network analysis revealed a highly interconnected symptom structure in which tolerance and withdrawal were the most central components, consistent with previous PPU network findings in Hungarian and Chinese samples (Chen et al., 2021). The prominence of these symptoms aligns with substance-related addiction frameworks in which escalation of engagement and withdrawal-related discomfort are proposed to contribute to the maintenance of addictive behaviors (Koob & Le Moal, 2001).

Gender-stratified networks, however, showed distinct structural patterns. In males, tolerance was the most central symptom, suggesting a greater role for escalation (in frequency and/or intensity) of pornography use, consistent with models emphasizing reward sensitivity, novelty seeking, and cue reactivity in male addiction trajectories (Mechelmans et al., 2014; Mitchell & Potenza, 2015; Voon et al., 2014). In contrast, the female network was more distributed, with withdrawal exerting the strongest influence and tolerance and salience also prominent, suggesting a greater involvement of negative-reinforcement and affect-regulation processes among women (Mitchell & Potenza, 2015). This interpretation is consistent with the stronger integration of mood modification within the female symptom network.

Additional differences emerged in the role of conflict. In the female network, conflict showed stronger connections with all other PPU symptom domains, most prominently with relapse and uniquely with salience, whereas this association was absent in males. One possible interpretation is that PPU among women in conservative sociocultural contexts may be more closely intertwined with moral incongruence and value-based distress (Eljawad et al., 2021; Grubbs & Perry, 2019; Mestre-Bach et al., 2021). By contrast, the strong tolerance–conflict relationship observed among men may reflect conflict arising primarily from the behavioral consequences of escalating use (Jiang et al., 2022).

The salience–relapse relationship also varied by gender, being absent in males but inversely associated in females. This divergence may stem from the cognitive-affective framing of the items. Salience in PPCS reflects reward-based anticipation (thinking about pornography, thinking about how good it would be, and planning to watch it), whereas relapse refers to failed abstinence attempts, which are often accompanied by negative self-appraisal (Bőthe et al., 2023; Mestre-Bach et al., 2021). Among individuals attempting to abstain due to guilt or value conflict, particularly females in restrictive cultural contexts, anticipatory cognitions may become decoupled from or even psychologically incompatible with relapse behavior. In contrast, relapse in males appeared more closely linked with withdrawal, tolerance, and mood modification, suggesting a pathway in which relapse is embedded within escalation and reinforcement cycles (Jiang et al., 2022; Koós et al., 2025). Future longitudinal or qualitative research could help clarify whether moral incongruence contributes to this pattern. Overall, these findings suggest that gender moderates both occurrence rate and symptom-network architecture, emphasizing the importance of cultural and gender-sensitive assessment and intervention.

### Implications for the Conceptualization and Classification of Problematic Pornography Use

The present findings also have implications for the conceptualization of PPU and for debates surrounding the broader classification of CSBD. A central issue in these discussions concerns whether problematic sexual behaviors should be conceptualized within an addiction framework or better understood through process-based accounts emphasizing motivational and regulatory mechanisms (Castro-Calvo et al., 2022; Sassover et al., 2022). Within this debate, particular attention has focused on the interpretation of addiction-related constructs such as tolerance and withdrawal, with some authors arguing that these symptoms may reflect normative variation in sexual desire rather than addictive pathology (Castro-Calvo et al., 2022).

In the present study, the six-factor structure of the PPCS replicated robustly in an Arabic-speaking sample, with tolerance and withdrawal emerging as central components within the symptom network. These findings suggest that the components described within Griffiths’ (2005) model capture core features of the symptom organization of PPU rather than functioning as peripheral indicators.

However, the gender-stratified networks indicated that similar symptom profiles may arise through different psychological pathways. Although the same six components were present across groups, their organization differed, with escalation-related dynamics appearing more prominent in males and affect-regulation processes more pronounced in females. This pattern suggests that the components described in behavioral addiction frameworks may capture a shared descriptive structure while being embedded within heterogeneous motivational and regulatory mechanisms (Castro-Calvo et al., 2022; Perales et al., 2020).

Collectively, these findings suggest that PPU warrants further consideration within an addiction framework while also highlighting the importance of integrating process-based perspectives to better understand variability in the mechanisms through which PPU develops and is maintained.

### Culturally Responsive Treatment Considerations

The validation of the Arabic PPCS supports its utility as a clinical and research tool for assessing PPU in Arabic-speaking populations and highlights potential targets for intervention. Previous research suggests that treatments addressing central symptoms such as tolerance and withdrawal may be more effective than approaches focusing solely on viewing frequency (Bőthe et al., 2020a, 2020b). The gender-stratified networks further suggest that treatment approaches may benefit from addressing different underlying dynamics. Among males, the prominence of escalation-related patterns may indicate the relevance of second-wave cognitive-behavioral approaches emphasizing cognitive restructuring, attentional-bias modification, cue-exposure, or urge-regulation techniques, and behavioral strategies that diversify reward (López-Pinar et al., 2025; Rodda et al., 2018). Among females, where affect-regulation processes appear most prominent, interventions focusing on emotional regulation and psychological flexibility, such as acceptance and commitment therapy, may be relevant (Lewczuk et al., 2017; López-Pinar et al., 2025; Twohig & Crosby, 2010; Yu et al., 2025). Complementary techniques, such as cognitive reappraisal, may also help mitigate withdrawal-related affect and self-critical appraisals that perpetuate use cycles (Wang et al., 2024).

In conservative sociocultural settings, such as Egypt, cultural adaptations may enhance treatment engagement and acceptability. Framing interventions around self-regulation, resilience, and personal values may reduce stigma-related barriers. Adaptations of cue-exposure or urge-regulation protocols using non-explicit symbolic cues may further improve acceptability (Hahn, 2025; Jalal et al., 2017). For individuals who view faith as central to their identity, religiously congruent coping strategies may complement clinical care. Practices such as dhikr (remembrance of God) and salah (prayer) may serve as attentional anchors or emotion regulation strategies within therapeutic frameworks. Other concepts such as Sabr (patience) or tawakkul (trust in God) may support coping during withdrawal-related distress. These adaptations should be offered as optional, patient-led components to preserve flexibility and respect for individual preferences (Jalal et al., 2017). Telehealth interventions may further improve access to care and reduce stigma-related barriers, particularly for women (Bőthe et al., 2021).

### Strengths, Limitations, and Future Directions

This study contributes a validated Arabic version of the PPCS, addressing an important gap in the assessment of PPU in Arabic-speaking populations. The large university sample enhanced statistical power, enabling robust analyses of gender invariance and symptom network structures within a conservative sociocultural context. The use of anonymous online data collection and social desirability measures further strengthens response validity when addressing a sensitive topic.

Nonetheless, several limitations should be considered. First, reliance on self-report measures introduces potential recall biases and self-presentation biases, despite statistical control for social desirability. Future research may benefit from incorporating objective or real-time approaches, such as ecological momentary assessment or implicit measures of motivational processes (Krumpal, 2013; Leighton et al., 2021). Second, the focus on Egyptian university students limits generalizability. Although this population is highly relevant given their high rates of digital media exposure, future studies should include community, clinical, and non-university samples across Arabic-speaking regions. The use of snowball sampling through social media may have introduced selection bias. However, this method also facilitated broad recruitment across diverse institutions and geographic areas (Leighton et al., 2021). Third, although the PPCS includes an empirically derived threshold score, its validity remains uncertain in Arabic-speaking populations.

Fourth, the inclusion criterion requiring pornography use at least once per week likely resulted in a sample biased toward more frequent users. Consequently, occurrence rate estimates should be interpreted cautiously. Future validation studies should include participants across a wider range of use frequencies and incorporate structured diagnostic interviews to better calibrate screening thresholds. Fifth, the cross-sectional design precludes causal inference regarding the relationships between PPU and psychological variables. Future longitudinal studies are needed to clarify the developmental course of PPU and the predictive role of specific symptoms over time. Finally, the PPCS operationalizes salience primarily in terms of positive anticipation and planning, which may not fully capture intrusive or unwanted pornography-related thoughts that may arise independently. Future studies should consider including non-appetitive salience items (e.g., “I experience unwanted thoughts about pornography”) to better reflect this dimension.

Beyond these methodological considerations, the findings also highlight opportunities for further conceptual development in PPU assessment. The PPCS captures the symptomatic structure of PPU but does not directly assess the psychological processes underlying these symptoms. Future instrument development may benefit from incorporating items that reflect process-level mechanisms emphasized in contemporary models of addictive behavior, including dissociations between motivational drive and experienced reward, diminished sensitivity to alternative rewards, the transition from impulsive to compulsive control, and the increasing reliance on pornography as a primary strategy for emotional regulation or stress relief (Castro-Calvo et al., 2022; Gola et al., 2017; Perales et al., 2020; Robinson & Berridge, 2025; Wang et al., 2024).

### Conclusion

The Arabic PPCS demonstrated strong psychometric properties and supports its use as a culturally appropriate instrument for assessing PPU among Arabic-speaking university students with regular pornography consumption. Network analyses identified gender-specific symptom patterns that may inform future work on culturally sensitive approaches and highlight how symptom-based addiction frameworks and process-level accounts can be integrated to advance the conceptualization and assessment of PPU across diverse populations.

## Supplementary Information

Below is the link to the electronic supplementary material.

Supplementary file1 (DOCX 1076 KB)

## Data Availability

The datasets generated and analyzed during the current study are available from the corresponding author upon reasonable request (r.abdoo.h2015@gmail.com) and are also openly accessible via the Open Science Framework at the following link: https://osf.io/853fh/?view_only=eb6a5d1f65844aa19474e0537efa722a
