# Supplementary material for: Arabic Validation of the Problematic Pornography Consumption Scale: Psychometric Properties, Measurement Invariance, and Gender-Specific Network Analysis
Source: Arch Sex Behav. 2026 Oct 14;55(7):3205–21. doi: 10.1007/s10508-026-03532-z (PMC13748031; doi:10.1007/s10508-026-03532-z)
Supplement: Supplementary file 1 [file 10508_2026_3532_MOESM1_ESM.docx]

# Section 1 (S1). Methods

## 1. Questionnaire completion time

We conducted a lab test on 10 individuals to estimate the minimum completion time to fill out the questionnaire. We found that the minimum time to fill out the questionnaire is 12 minutes, and 9 minutes is the minimum time to complete the questionnaire without participating in PPCS. Any participant who completed the whole questionnaire in less than 9 minutes was excluded from the analysis.

## 2. Translation and Cultural Adaptation Details for the Arabic PPCS

### 2.1.  Adaptation philosophy and process overview

The development of the Arabic version of the Problematic Pornography Consumption Scale (PPCS) adhered to the International Test Commission (ITC) guidelines for test adaptation (Commission, 2017). The process involved a standard methodology including initial forward translation from English to Arabic by bilingual experts, synthesis of these translations, back-translation into English by an independent translator blind to the original scale, and review by an expert committee comprising psychologists, psychiatrists, and linguists familiar with both the construct and the target Egyptian culture. The primary goal throughout was to achieve conceptual and functional equivalence rather than a strict literal translation, ensuring the items accurately capture the intended underlying constructs of PPU while using language that is natural, clear, and culturally resonant for Egyptian university students. The resulting pre-final version underwent pilot testing (n=25) to assess clarity, comprehension, and cultural appropriateness, with feedback informing the final item wording presented herein.

### 2.2.  Specific linguistic and cultural adaptations

While many items allowed for reasonably direct translation, several required careful adaptations to maximize clarity and cultural suitability for Arabic speakers:

**Phrasing for Naturalness:** In some instances, direct translation of English phrasing could result in slightly unnatural or stilted Arabic. For example, Item 2 ("I used porn to restore the tranquility of my feelings") was adapted to "استخدمت المواد الإباحية لتهدئة مشاعري" (Ustakhdamtu al-mawad al-ibahiyya li-tahdi'at mashaeiri), meaning "I used pornography to calm my feelings," which is a more common and direct way to express seeking emotional soothing in Arabic. Similarly, Item 8 ("Watching porn got rid of my negative feelings") became "مشاهدة المواد الإباحية كانت وسيلة للتخلص من المشاعر السلبية لدي" (Mushahadat al-mawad al-ibahiyya kanat wasilatan li-takhalus min al-mashaeir al-salbiyya ladayya), translating to "Watching pornography was a way/means for me to get rid of negative feelings," providing slightly more standard phrasing.

**Lexical Choices for Contextual Fit:** Word choice was critical. For instance, in Item 10 ("I felt that I needed more and more porn in order to satisfy my needs"), the word "needs" was translated as "رغباتي" (raghabati - desires) rather than the more literal "احتياجاتي" (ihtiyajati - needs). In the context of pornography consumption and satisfaction, "desires" carries a more fitting connotation for Arabic speakers than the broader term "needs."

**Nuances in Emotional/Internal States:** Terms describing internal states were chosen carefully. "Became stressed" (Item 6) was rendered as "أشعر بالتوتر" (Ash'uru bi-tawattur - I feel stressed/tense), using a common expression. "Became agitated" (Item 12) was translated as "شعرت بالاضطراب" (Sha'artu bi-idtirab - I felt disturbed/agitated), selecting a term that captures the intended negative arousal state effectively in Arabic.

**Maintaining Construct Meaning:** Across all items, the focus remained on ensuring the core meaning related to the specific component of Griffiths' model (salience, mood modification, tolerance, withdrawal, conflict, relapse) was accurately conveyed, even if the exact surface wording differed slightly from the English original.

### 2.3.  Rationale for adaptations

These adaptations were deemed necessary by the expert committee and informed by pilot feedback to avoid potential misunderstandings, reduce cognitive load for participants, and ensure the scale felt linguistically appropriate rather than like a foreign translation. By prioritizing natural Arabic phrasing and contextually suitable vocabulary, we aimed to produce a final version of the Arabic PPCS that not just Egyptian university students but also all Arabic speakers could readily comprehend and respond to accurately, thereby enhancing the validity of the collected data for this population. The successful psychometric results reported in the main manuscript support the effectiveness of this adaptation approach.

**English and Arabic versions of the Problematic Pornography Consumption Scale (PPCS)**

(Bőthe et al., 2018)

Please think back to the past six months and indicate on the following 7-point scale how often or to what extent the statements apply to you. There is no right or wrong answer. Please indicate the answer that most applies to you.

مقياس استهلاك المواد الإباحية الإشكالي (PPCS)

يرجى الرجوع إلى الأشهر الستة الماضية والإشارة على المقياس التالي المكون من 7 نقاط إلى عدد المرات أو إلى أي مدى تنطبق العبارات عليك. ليس هناك جواب صحيح أو خاطئ. يرجى الإشارة إلى الإجابة التي تنطبق عليك أكثر.

صَّممته الباحثة َ Bőthe وُزملاؤها عام 2018م، يُقِّيم مشكلة استهلاك المواد الإباحَّية باستخدام نموذج إدمان Griffiths, 2005 المكَّون من 18 عبارة، ُمقَسم إلى ستة مكونات لقياس:

1. **بروز المشكلة (الاستحواذ الفكري)** ، والتي تعني مدى أهمية استهلاك المواد الإباحَّية ومدى َهْيمنتها في يوميات الفرد، ويتكَّون من 3عبارات.
2. **مكِّون التأثير على المزاج**، والذي يقيس مدى تحسن مزاج ُمستهلك المواد الإباحية حين استهلاكها، ويتكَّون من 3عبارات.
3. **الصراع**، ويقيس الصراعات داخل النفس والنزاعات الُمهيمنة، ويتكَّون من 3عبارات.
4. **التحُّمل**، ويقيس العمليات التي تتطَّلب نشاطات متزايدة لتحقيق نفس تأثيرات تعديل الحالة المزاجية، ويتكَّون من 3عبارات.
5. **مكِّون الانتكاس**، والذي يقيس الانتكاسات المتكِّررة للأنماط السابقة، لاستخدام المواد الإباحَّية ومدى البُعد الزمني بين محاولات التوقف والامتناع عن استهلاك المواد الإباحية، ويتكَّون من 3عبارات.
6. **مكِّون الانسحاب**، والذي يقيس المشاعر غير السارة والحالات العاطفية التي تحدث عندما يتوقف نشاط استهلاك المواد الإباحية، ويتكَّون من 3عبارات.

يُصحح المقياس على مقياس ليكرت ،7حيث تتفاوت الإجابات من 1 حتى 7، بحيث تُعطى العبارة رقم 7في حال كانت العبارة تنطبق رقم في كل الأحوال وتُعطى العبارة 1في حال كانت العبارة لا تنطبق أبًدا.

| شعرت بأن المواد الإباحية هي جزء مهم من حياتي. | 1. I felt that porn is an important part of my life |
| --- | --- |
| استخدمت المواد الإباحية لتهدئة مشاعري. | 2. I used porn to restore the tranquility of my feelings |
| شعرت بأن المواد الإباحية تتسببت في مشاكل في حياتي الجنسية. | 3. I felt porn caused problems in my sexual life |
| شعرت بأنني بحاجة لمشاهدة المزيد والمزيد من المواد الإباحية للشعور بالرضا. | 4. I felt that I had to watch more and more porn for satisfaction |
| حاولت دون جدوى تقليل كمية المواد الإباحية التي أشاهدها. | 5.I unsuccessfully tried to reduce the amount of porn I watch |
| أشعر بالتوتر عندما يمنعني شيء ما من مشاهدة المواد الإباحية. | 6. I became stressed when something prevented me from watching porn |
| فكرت في مدى روعة مشاهدة المواد الإباحية. | 7. I thought about how good it would be to watch porn |
| مشاهدة المواد الإباحية كانت وسيلة للتخلص من المشاعر السلبية لدي. | 8. Watching porn got rid of my negative feelings |
| منعتني مشاهدة المواد الإباحية من تقديم أفضل ما لدي. | 9. Watching porn prevented me from bringing out the best in me |
| شعرت بأنني بحاجة إلى المزيد والمزيد من المواد الإباحية من أجل إشباع رغباتي. | 10. I felt that I needed more and more porn in order to satisfy my needs |
| عندما تعهدت بعدم مشاهدة المواد الإباحية بعد الآن، لم أستطع فعل ذلك إلا لفترة قصيرة من الزمن. | 11. When I vowed not to watch porn anymore, I could only do it for a short period of time |
| شعرت بالاضطراب عندما لم أتمكن من مشاهدة المواد الإباحية. | 12. I became agitated when I was unable to watch porn |
| كنت أخطط باستمرار متى سأشاهد المواد الإباحية. | 13. I continually planned when to watch porn |
| تخلصت من توتري من خلال مشاهدة المواد الإباحية. | 14. I released my tension by watching porn |
| أهملت الأنشطة الترفيهية الأخرى نتيجة لمشاهدة المواد الإباحية. | 15. I neglected other leisure activities as a result of watching porn |
| شاهدت تدريجيًا المزيد من المواد الإباحية "المتطرفة"، لأن المواد الإباحية التي شاهدتها من قبل أصبحت أقل إرضاءً أو إشباعًا. | 16. I gradually watched more "extreme" porn, because the porn I watched before was less satisfying |
| قاومت مشاهدة المواد الإباحية لفترة قصيرة فقط قبل أن أتعرض للانتكاس. | 17. I resisted watching porn for only a little while before I relapsed |
| افتقدت المواد الإباحية كثيرًا عندما لم أشاهدها لفترة. | 18. I missed porn greatly when I didn't watch it for a while |

| الرقم | الاختيار | الترجمة العربية |
| --- | --- | --- |
| 1 | Never | أبداً |
| 2 | Rarely | نادراً |
| 3 | Occasionally | أحياناً قليلة |
| 4 | Sometimes | أحياناً |
| 5 | Often | غالباً |
| 6 | Very Often | غالباً جداً |
| 7 | All the Time | طوال الوقت |

Scoring

Add the scores of the items of each factor. For the total score add all the scores of the items. 76 points or more indicate possible problematic pornography use

**Salience**: 1, 7, 13

Mood modification: 2, 8, 14

**Conflict**: 3, 9, 15

**Tolerance**: 4, 10, 16

**Relapse**: 5, 11, 17

**Withdrawal**: 6, 12, 18

مفتاح التصحيح:

أضف درجات عناصر كل عامل. للحصول على النتيجة الإجمالية أضف جميع درجات العناصر. تشير 76 نقطة أو أكثر إلى احتمال استخدام المواد الإباحية الإشكالية.

البروز (الاستحواذ الفكري(: 1، 7، 13

تشير إلى مدى سيطرة التفكير في المحتوى الإباحي على حياة الشخص

مكون التأثيرعلى المزاج : 2،8، 14

تتعلق باستخدام المحتوى الإباحي للتأثير على الحالة المزاجية أو المشاعر

الصراع: 3، 9، 15

يشير إلى التعارض بين استخدام المحتوى الإباحي والجوانب الأخرى من حياة الشخص

التحمل : 4، 10، 16

يتعلق بالحاجة المتزايدة لاستهلاك المزيد من المحتوى الإباحي أو لفترات أطول للحصول على نفس التأثير

مكون الانتكاس: 5، 11، 17

يشير إلى العودة لأنماط الاستخدام السابقة بعد فترة من التوقف أو التقليل

مكون الانسحاب : 6، 12، 18

يتعلق بالأعراض النفسية أو الجسدية السلبية عند التوقف عن استخدام المحتوى الإباحي

# Section 2 (S2). Results

# 1.  Results of Item-to-Item Analysis

Item-to-item correlation analysis revealed strong intercorrelations among all 18 items of the Arabic PPCS, with correlation coefficients ranging from r = 0.287 to r = 0.758 (all p < .001). The strongest correlations were observed between Q10 and Q4 (r = 0.758), Q12 and Q6 (r = 0.735), and Q17 and Q11 (r = 0.722), suggesting particularly robust relationships between these item pairs. The lowest, though still significant, correlation was between Q14 and Q3 (r = 0.287). These strong intercorrelations support the internal coherence of the scale, indicating that the items consistently measure related aspects of problematic pornography consumption. The pattern of correlations also aligns with the theoretical six-factor structure, as items within the same subscale generally showed stronger associations with each other than with items from different subscales. For example, items Q10, Q11, and Q12 (representing the Tolerance subscale) demonstrated high intercorrelations, as did items Q16, Q17, and Q18 (representing the Withdrawal subscale). The comprehensive correlation matrix (Table S1) provides evidence for the robust internal structure of the Arabic PPCS, supporting its reliability as a measure of problematic pornography consumption in this population

| variables | Q1 | Q2 | Q3 | Q4 | Q5 | Q6 | Q7 | Q8 | Q9 | Q10 | Q11 | Q12 | Q13 | Q14 | Q15 | Q16 | Q17 | Q 18 |
| --- | --- | --- | --- | --- | --- | --- | --- | --- | --- | --- | --- | --- | --- | --- | --- | --- | --- | --- |
| Q1 | 1 |  |  |  |  |  |  |  |  |  |  |  |  |  |  |  |  |  |
| Q2 | .609*** | 1 |  |  |  |  |  |  |  |  |  |  |  |  |  |  |  |  |
| Q3 | .343*** | .396 *** | 1 |  |  |  |  |  |  |  |  |  |  |  |  |  |  |  |
| Q4 | .569*** | .623  *** | .422*** | 1 |  |  |  |  |  |  |  |  |  |  |  |  |  |  |
| Q5 | .367*** | .444 *** | .357*** | .504  *** | 1 |  |  |  |  |  |  |  |  |  |  |  |  |  |
| Q6 | .523*** | .513  *** | .409*** | .597  *** | .411  *** | 1 |  |  |  |  |  |  |  |  |  |  |  |  |
| Q7 | .511*** | .467  *** | .308  *** | .570  *** | .347  *** | .581  *** | 1 |  |  |  |  |  |  |  |  |  |  |  |
| Q8 | .526*** | .720  *** | .338  *** | .586  *** | .452  *** | .524  *** | .503  *** | 1 |  |  |  |  |  |  |  |  |  |  |
| Q9 | .354*** | .447  *** | .482  *** | .530  *** | .523  *** | .490  *** | .343  *** | .461  *** | 1 |  |  |  |  |  |  |  |  |  |
| Q10 | .544*** | .602  *** | .379  *** | .758  *** | .497  *** | .610  *** | .604  *** | .603  *** | .574  *** | 1 |  |  |  |  |  |  |  |  |
| Q11 | .404*** | .470  *** | .343  *** | .524  *** | .676  *** | .434  *** | .380  *** | .483  *** | .553  *** | .568  *** | 1 |  |  |  |  |  |  |  |
| Q12 | .521*** | .536  *** | .427  *** | .613  *** | .440  *** | .735  *** | .551  *** | .550  *** | .534  *** | .673  *** | .528  *** | 1 |  |  |  |  |  |  |
| Q13 | .536*** | .493  *** | .374  *** | .542  *** | .351  *** | .594  *** | .556  *** | .476  *** | .388  *** | .572  *** | .418  *** | .618  *** | 1 |  |  |  |  |  |
| Q14 | .468*** | .598  *** | .287  *** | .495  *** | .338  *** | .501  *** | .502  *** | .633  *** | .322  *** | .517  *** | .380  *** | .544  *** | .564  *** | 1 |  |  |  |  |
| Q15 | .445*** | .494  *** | .446  *** | .579  *** | .450  *** | .559  *** | .474  *** | .513  *** | .607  *** | .617  *** | .487  *** | .597  *** | .539  *** | .487  *** | 1 |  |  |  |
| Q16 | .421*** | .492  *** | .378  *** | .548  *** | .420  *** | .504  *** | .491  *** | .520  *** | .470  *** | .603  *** | .492  *** | .531  *** | .508  *** | .450  *** | .554  *** | 1 |  |  |
| Q17 | .365*** | .469  *** | .335  *** | .516  *** | .648  *** | .423  *** | .385  *** | .501  *** | .564  *** | .546  *** | .722  *** | .487  *** | .385  *** | .381  *** | .511  *** | .522  *** | 1 |  |
| Q18 | .542*** | .578  *** | .366  *** | .640  *** | .474  *** | .602  *** | .588  *** | .553  *** | .464  *** | .669  *** | .553  *** | .651  *** | .625  *** | .549  *** | .535  *** | .557  *** | .567  *** | 1 |
| **Table S1**: Item-to-Item correlation analysis, *** Correlation is significant at *p***<** .001 level (2-tailed). | | | | | | | | | | | | | | | | | | |

## 2. Confirmatory Factor Analysis

Confirmatory factor analysis (CFA) was conducted to validate the six-factor structure of the Arabic PPCS. The analysis revealed strong and statistically significant factor loadings for all items on their respective factors (all p < .001), as shown in Table S2. The factor loadings ranged from 0.588 to 0.890, indicating moderate to strong relationships between each item and its corresponding factor. The Mood Modification factor showed particularly robust loadings, with Q2 loading at 0.859 and Q8 at 0.853. The Tolerance factor also demonstrated strong loadings, with Q10 having the highest loading on the entire scale at 0.890, followed by Q4 at 0.859. The Conflict factor contained the lowest loading item (Q3 at 0.588), though this value still represents an acceptable loading. The Salience factor items (Q1, Q7, Q13) showed consistent loadings ranging from 0.750 to 0.783. The Relapse factor items (Q5, Q11, Q17) demonstrated strong loadings between 0.783 and 0.864, with Q5 having the highest loading. The Withdrawal factor items (Q6, Q12, Q18) also showed robust loadings ranging from 0.814 to 0.854. These results provide strong support for the six-factor structure of the Arabic PPCS, confirming that the translated scale maintains the same dimensional structure as the original instrument. The significant loadings across all factors indicate that the Arabic version effectively captures the multidimensional nature of problematic pornography consumption as conceptualized in Griffiths' components model of addiction

| ***Table S2: Confirmatory factor analysis loadings*** | |
| --- | --- |
| **Factor** | **Questions (Loading)** |
| **Salience** | Q1 (0.750***)  Q7 (0.778***)  Q13 (0.783***) |
| **Mood Modification** | Q2 (0.859***)  Q8 (0.853***)  Q14 (0.769***) |
| **Conflict** | Q3 (0.588***)  Q9 (0.771***)  Q15 (0.818***) |
| **Tolerance** | Q4 (0.859***)  Q10 (0.890***)  Q16 (0.726***) |
| **Relapse** | Q5 (0.864***)  Q11 (0.783***)  Q17 (0.842***) |
| **Withdrawal** | Q6 (0.830***)  Q12 (0.854***)  Q18 (0.814***) |
| Note. *** p-value < 0.001 | |
|  | |

## 3. Comparison between crude correlation and partial correlations

| **Table S3. The table shows a comparison** | | | | |
| --- | --- | --- | --- | --- |
| **Variable Pair** | **Crude Spearman's ρ** | **Partial ρ (Controlled)** | **% Change** | **Significance (Both)** |
| Internet - PPU | 0.491 | 0.45** | -8.4% | p < .001 |
| Anxiety - PPU | 0.413 | 0.37** | -10.4% | p < .001 |
| Depression - PPU. | 0.438 | 0.42** | -4.1% | p < .001 |
| Stress - PPU | 0.378 | 0.44** | +16.4% | p < .001 |

4. **Network Bootstrapping analyses**
   4.1. *Edge Stability Analysis*

Non-parametric bootstrap analyses were conducted to assess the stability of edge weights in the symptom networks of problematic pornography use, estimated separately for male and female participants. The results, illustrated in Figure S1, show that for both male and female networks, several edges demonstrated high stability, as indicated by narrow 95% confidence intervals (CIs) that did not encompass zero, suggesting robust associations.

In the male network, the edge between Salience and Withdrawal exhibited the highest stability, followed by Tolerance–Withdrawal, Conflict–Tolerance, and Mood Modification–Withdrawal. These connections showed narrow confidence intervals and minimal deviation between the original edge estimates (black dots) and the bootstrap means (red dots), indicating consistency across resampled networks. Conversely, edges such as Salience–Relapse and Salience–Conflict displayed wider CIs overlapping with zero, suggesting lower robustness and limited replicability.

In the female network, the most stable edges were observed between Tolerance and Withdrawal, Conflict–Relapse, and Mood Modification–Tolerance. These edges demonstrated both higher mean weights and narrower confidence intervals. Similar to the male network, less stable connections, such as Salience–Relapse and Relapse–Withdrawal, showed wide CIs that encompassed zero, indicating variability and insufficient evidence for a robust association in these connections.

Overall, these findings support the presence of core, stable symptom interactions (e.g., Withdrawal, Tolerance, and Mood Modification connections) in both networks, while highlighting differences in the pattern and strength of edge stability between male and female participants.

4.2. *Centrality Stability Analysis*

Centrality stability analyses were conducted to evaluate the robustness of node-level importance within the network models of problematic pornography use. Three centrality metrics—strength, betweenness, and closeness—were estimated and tested for stability using non-parametric bootstrapping with 1000 resamples (**Figure S2**). The analysis was performed separately for male and female participants.

In the male network, the node Tolerance demonstrated the highest strength centrality (1.20), with moderate stability reflected by several significant deviations from its bootstrapped distribution (black boxes). Other nodes, such as Mood Modification (0.77) and Conflict (0.76), also exhibited high strength but with signs of instability, particularly in betweenness and closeness centrality. Betweenness centrality was highest for Tolerance (12.0) and Withdrawal (8.0), but these values lacked stability as indicated by their widespread significant deviations across node comparisons. Closeness centrality values were relatively low across all nodes and generally unstable.

In the female network, Tolerance (1.10), Mood Modification (0.85), and Conflict (0.83) showed the highest strength centrality values, with slightly greater stability compared to the male network. However, black boxes across several comparisons still indicate partial instability in these estimates. Betweenness centrality was highest for Withdrawal (8.0) and Tolerance (6.0), mirroring the male network pattern, but again, these estimates showed low robustness. Closeness centrality values remained low for all nodes, with limited stability

**Figure S1. Edge Stability of Network Connections for Male and Female Participants.**
This figure illustrates the stability of the estimated edge weights (partial correlations) for the problematic pornography use networks, separately for male (left panel) and female (right panel) participants, derived from non-parametric bootstrapping with 1000 resamples. Each row represents a specific edge (connection between two factors). The x-axis indicates the edge weight value. The black dots represent the edge weight estimate from the original sample. The red dots indicate the mean edge weight across the bootstrap samples. The grey shaded area surrounding the line of dots represents the 95% confidence interval for each edge weight. Wider grey areas indicate greater variability and less stability for that particular edge weight; if the grey area encompasses zero, the presence or strength of the edge is considered not robust

**Figure S2. Centrality Stability of Network Nodes for Male and Female Participants.**
This figure presents the stability of three centrality measures—Strength, Betweenness, and Closeness—for each of the six network nodes (Tolerance, Withdrawal, Mood Modification, Conflict, Relapse, Salience), separately for male (left panel) and female (right panel) participants. The stability is assessed by comparing the centrality values from the original sample to their distributions derived from non-parametric bootstrapping with 1000 resamples. Within each panel and for each centrality measure, rows represent the network nodes, and columns (beneath Strength, Betweenness, and Closeness headers) also represent the network nodes for comparison purposes. Black boxes indicate that the centrality measure of the node listed in the row was found to be significantly different (e.g., at an alpha of 0.05) from its bootstrapped distribution when compared against the node listed in the column, suggesting potential instability in its estimated centrality value. Grey boxes indicate no significant difference, implying greater stability. The numerical values overlaid on the plot represent the centrality estimates from the original sample for the node listed in the row.

**Section 3 (S3). The Confirmatory Analysis of The Psychometric Validation of Arabic PPCS**

To confirm the Psychometric validity results of the Arabic PPCs-18. We conducted a sub-sample analysis of 494 participants, who were randomly selected.

1. **Sample Characteristics**

A total of 494 university students participated in the study. The sample was predominantly male (68.2%) and Egyptian (92.7%), with most participants studying in health-related fields (60.5%) and residing in urban areas (58.1%). Students were distributed across academic years, with the majority in the first, second, or third year. Nearly one in six participants (15.8%) reported a family history of psychiatric illness, and 10.9% reported having a chronic disease. The majority were single (98.4%). Approximately 23.1% met criteria for problematic pornography consumption risk. Full demographic details are presented in Table S3.

2. **Reliability Analysis**

The Problematic Pornography Consumption Scale (PPCS) demonstrated excellent internal consistency, with Cronbach’s alpha of 0.9 for the overall scale. Item-to-total score correlations ranged from 0.640 to 0.864, indicating that all items contributed substantially to the total scale score. (**Table S4)**

Meanwhile, the item-to-item correlation matrix revealed significant positive correlations (p < .001) among all items, ranging from 0.379 (between items Q14 and Q3) to 0.834 (between items Q6 and Q12). These correlations provide further evidence of the scale's internal consistency and the interrelatedness of the items within the PPCS. (Table S5)

Confirmatory factor analysis (CFA) was also conducted to evaluate the factor structure of the PPCS. The chi-square test indicated a significant difference between the model and the observed data (Χ² (120) = 419.094, p < .001). However, given the large sample size, this result is expected, and other fit indices were examined to assess model fit. The CFA model demonstrated an excellent fit to the data, as indicated by the following fit indices: Comparative Fit Index (CFI) = 0.988, Tucker-Lewis Index (TLI) = 0.985, Bentler-Bonett Normed Fit Index (NFI) = 0.984, Parsimony Normed Fit Index (PNFI) = 0.783, Root Mean Square Error of Approximation (RMSEA) = 0.071 with a 90% confidence interval of 0.064 to 0.079, and Standardized Root Mean Square Residual (SRMR) = 0.028. The Kaiser-Meyer-Olkin (KMO) measure of sampling adequacy was 0.956, and Bartlett's Test of Sphericity was significant (χ² (153) = 10082.910, p < .001), indicating that the data were suitable for factor analysis. The results supported the six-factor model proposed by the scale developers, with all items loading significantly on their respective factors (p < .001). The factor loadings ranged from 0.720 (item Q3 on the Conflict factor) to 0.953 (item Q15 on the Conflict factor). (**Table S4)**

To evaluate the measurement invariance of the Arabic version of the Problematic Pornography Consumption Scale (PPCS) across gender, a stepwise multi-group confirmatory factor analysis (CFA) was conducted. The **configural model**, which tested whether the same factor structure held for both males and females without constraints, showed good model fit: χ²(252) = 665.65, CFI = 0.988, TLI = 0.985, RMSEA = 0.059, SRMR = 0.038. The **metric model**, which constrained factor loadings to be equal across groups, also demonstrated acceptable fit: χ²(336) = 861.59, CFI = 0.985, TLI = 0.986, RMSEA = 0.057, SRMR = 0.035. The change in fit indices from the configural model (ΔCFI = -0.003, ΔRMSEA = -0.002) was within acceptable thresholds (ΔCFI < 0.01, ΔRMSEA < 0.015), supporting metric invariance. The **scalar model**, in which both factor loadings and item intercepts were constrained to be equal across genders, also indicated good fit: χ²(336) = 572.60, CFI = 0.991, TLI = 0.992, RMSEA = 0.054, SRMR = 0.034. Compared to the metric model, ΔCFI was +0.006 and ΔRMSEA was -0.003, supporting scalar invariance. (Table S6)

3. **Construct Validity**

PPCS-18 total scores showed moderate to strong positive correlations with GAD-7 (r = 0.459), PSS-10 (r = 0.441), PHQ-9 (r = 0.489), and IAT-20 (r = 0.524) scores (all p < .001). These correlations are visualized in **Figure S3**

**Partial Spearman's rho heatmap**

**Figure S1. Spearman partial correlation Heatmap of the study's main variables, controlling for gender, Field of study, Area of housing, family history of psychological illness, sleeping hours, social desirability, and chronic diseases.**

4. **Network Analysis by Gender**

Network analysis was conducted separately for males and females to examine the interrelations among the six PPCS factors: salience (S), mood modification (M), conflict (C), tolerance (T), withdrawal (W), and relapse (R). Both networks demonstrated high connectivity, with 14 non-zero edges for males (sparsity = 0.067) and 13 for females (sparsity = 0.133). Centrality indices revealed notable gender differences. Among males, **tolerance** exhibited the highest centrality across all measures, particularly in betweenness (2.041) and strength (1.383), indicating a key bridging and influential role in the male network. In contrast, among females, **withdrawal** showed the highest strength (0.981) and expected influence (0.981), followed by tolerance. These findings support the results of the main sample that tolerance plays a central role in male problematic pornography use patterns, while withdrawal symptoms are more central in the female network structure. Figure S2 shows the network plots, while Figure S5 shows the centrality plots

**Figure S4. Network Plots of females and males**

**Female**

**Male**

Figure S5. Centrality plots

| **Table S3. Sample Characteristics (N = 494)** | | | |
| --- | --- | --- | --- |
| **Variable** | **Category** | **n** | **%** |
| **Sex** | | | |
|  | Male | 337 | 68.2% |
|  | Female | 157 | 31.8% |
| **Field of Study** | | | |
|  | Health-related | 299 | 60.5% |
|  | Non-health-related | 195 | 39.5% |
| **Year of School** | | | |
|  | First | 106 | 21.5% |
|  | Second | 124 | 25.1% |
|  | Third | 93 | 18.8% |
|  | Fourth | 81 | 16.4% |
|  | Fifth | 52 | 10.5% |
|  | Sixth | 38 | 7.7% |
| **Region of Residence** | | | |
|  | Greater Cairo and Delta | 236 | 47.8% |
|  | Alexandria (ALEX) | 134 | 27.1% |
|  | Canal | 34 | 6.9% |
|  | Upper Egypt | 90 | 18.2% |
| **Nationality** | | | |
|  | Egyptian | 458 | 92.7% |
|  | Non-Egyptian | 36 | 7.3% |
| **Area of Housing** | | | |
|  | Rural | 207 | 41.9% |
|  | Urban (City) | 287 | 58.1% |
| **Family History of Psychiatric Illness** | | | |
|  | Yes | 78 | 15.8% |
|  | No | 416 | 84.2% |
| **Chronic Illness** | | | |
|  | Yes | 54 | 10.9% |
|  | No | 440 | 89.1% |
| **Marital Status** | | | |
|  | Single | 486 | 98.4% |
|  | Married | 5 | 1.0% |
|  | Divorced | 3 | 0.6% |
| **Problematic Pornography consumption** | | | |
|  | Normal | 380 | 76.9% |
|  | Risk Problematic Use | 114 | 23.1% |
| **Q1. Academic Motivation**: *I always strive to achieve the highest academic grades so that I gain the appreciation of the people around me* | | | |
|  | Strongly disagree | 104 | 21.1% |
|  | Disagree | 107 | 21.7% |
|  | Neutral | 157 | 31.8% |
|  | Agree | 89 | 18.0% |
|  | Strongly agree | 37 | 7.5% |
| **Q2. Honesty**: *When I don’t know something, I have no objection to admitting it* | | | |
|  | Strongly disagree | 4 | 0.8% |
|  | Disagree | 21 | 4.3% |
|  | Neutral | 50 | 10.1% |
|  | Agree | 189 | 38.3% |
|  | Strongly agree | 230 | 46.6% |
| **Social Desirability Answering** | | | |
|  | Answering in a socially desirable way | 176 | 35.4% |
|  | Answering in a non-socially desirable way | 318 | 64.4% |
|  | | | |

|  | **Table S4. Item to total and item to rest analyses results and CFA analysis results** | | | | | | | |  |
| --- | --- | --- | --- | --- | --- | --- | --- | --- | --- |
|  | **variables** | **Item-to-Total Score Correlation** | **Factor loadings of CFA** | | | | | |  |
|  |  |  | ***Salience factor*** | ***Mood modification*** | ***Conflict factor*** | ***Tolerance factor*** | ***Relapse factor*** | ***Withdrawal factor*** |  |
|  | Q1 | .772^**^ | **0.825***** |  |  |  |  |  |  |
|  | Q2 | .833^**^ |  | 0.929******* |  |  |  |  |  |
|  | Q3 | .640^**^ |  |  | 0.720******* |  |  |  |  |
|  | Q4 | .864^**^ |  |  |  | 0.922******* |  |  |  |
|  | Q5 | .738^**^ |  |  |  |  | 0.878******* |  |  |
|  | Q6 | .829^**^ |  |  |  |  |  | 0.932******* |  |
|  | Q7 | .779^**^ | 0.845****** |  |  |  |  |  |  |
|  | Q8 | .797^**^ |  | 0.895******* |  |  |  |  |  |
|  | Q9 | .724^**^ |  |  | 0.847******* |  |  |  |  |
|  | Q10 | .863^**^ |  |  |  | 0.931******* |  |  |  |
|  | Q11 | .763^**^ |  |  |  |  | 0.899******* |  |  |
|  | Q12 | .848^**^ |  |  |  |  |  | 0.932******* |  |
|  | Q13 | .801^**^ | 0.890******* |  |  |  |  |  |  |
|  | Q14 | .733^**^ |  | 0.847******* |  |  |  |  |  |
|  | Q15 | .830^**^ |  |  | 0.953******* |  |  |  |  |
|  | Q16 | .765^**^ |  |  |  | 0.800******* |  |  |  |
|  | Q17 | .764^**^ |  |  |  |  | 0.892******* |  |  |
|  | Q18 | .852^**^ |  |  |  |  |  | 0.911******* |  |
|  | **CFA Indices** | | | | | | | |  |
|  | **(CFI)** | 0.988 | | | | | | |  |
|  | **(TLI)** | 0.985 | | | | | | |  |
|  | **(NFI)** | 0.984 | | | | | | |  |
|  | **PNFI)** | 0.783 | | | | | | |  |
|  | **(RMSEA)[CI]** | 0.071 **[0.064: 0.079]** | | | | | | |  |
|  | **(SRMR)** | 0.028 | | | | | | |  |
|  | **(KMO) Test** | 0.956 | | | | | | |  |
|  | **Bartlett's Test** | (Χ² (153) = 10082.910, p **< .001**) | | | | | | |  |
|  |  | | | | | | | |  |
| ***Note***, this table contains factor loadings and the fit indices for the Confirmatory Factor Analysis (CFA) of the Arabic PPCS-18, assessing the six-factor structure. Model fit was evaluated using multiple indices. The Chi-square (χ²) test assesses exact model fit, with lower values indicating better fit. The Comparative Fit Index (CFI) and Tucker-Lewis Index (TLI) values of ≥ 0.95 suggest a good model fit. Similarly, the Normed Fit Index (NFI) and Parsimony Normed Fit Index (PNFI) provide measures of relative fit, where higher values indicate better model performance. The Root Mean Square Error of Approximation (RMSEA) values of ≤ 0.08 suggest an acceptable fit, while values of ≤ 0.06 indicate a good fit. The Standardized Root Mean Square Residual (SRMR) values of ≤ 0.08 further confirm an adequate fit. Sampling adequacy was assessed using the Kaiser-Meyer-Olkin (KMO) test, where values > 0.90 indicate excellent sampling adequacy. Finally, Bartlett’s test of sphericity, with a significant p-value (p < 0.001), confirmed that the data were appropriate for factor analysis. Overall, these indices suggest that the six-factor structure of the PPCS-18 demonstrates an acceptable model fit. ***, p < .001*.* | | | | | | | | | |

| **Table S5. Item to Item analysis results** | | | | | | | | | | | | | | | | | | |
| --- | --- | --- | --- | --- | --- | --- | --- | --- | --- | --- | --- | --- | --- | --- | --- | --- | --- | --- |
| variables | **Q1** | **Q2** | **Q3** | **Q4** | **Q5** | **Q6** | **Q7** | **Q8** | **Q9** | **Q**  **10** | **Q**  **11** | **Q**  **12** | **Q**  **13** | **Q**  **14** | **Q**  **15** | **Q**  **16** | **Q**  **17** | **Q18** |
| Q1 | 1 |  |  |  |  |  |  |  |  |  |  |  |  |  |  |  |  |  |
| Q2 | .739^***^ | 1 |  |  |  |  |  |  |  |  |  |  |  |  |  |  |  |  |
| Q3 | .478^***^ | .511^***^ | 1 |  |  |  |  |  |  |  |  |  |  |  |  |  |  |  |
| Q4 | .689^***^ | .739^***^ | .517^***^ | 1 |  |  |  |  |  |  |  |  |  |  |  |  |  |  |
| Q5 | .512^***^ | .547^***^ | .404^***^ | .585^***^ | 1 |  |  |  |  |  |  |  |  |  |  |  |  |  |
| Q6 | .672^***^ | .660^***^ | .504^***^ | .712^***^ | .511^***^ | 1 |  |  |  |  |  |  |  |  |  |  |  |  |
| Q7 | .652^***^ | .600^***^ | .416^***^ | .692^***^ | .481^***^ | .690^***^ | 1 |  |  |  |  |  |  |  |  |  |  |  |
| Q8 | .606^***^ | .789^***^ | .442^***^ | .661^***^ | .553^***^ | .617^***^ | .592^***^ | 1 |  |  |  |  |  |  |  |  |  |  |
| Q9 | .456^***^ | .497^***^ | .555^***^ | .579^***^ | .571^***^ | .551^***^ | .448^***^ | .489^***^ | 1 |  |  |  |  |  |  |  |  |  |
| Q10 | .634^***^ | .688^***^ | .456^***^ | .826^***^ | .599^***^ | .695^***^ | .725^***^ | .661^***^ | .616^***^ | 1 |  |  |  |  |  |  |  |  |
| Q11 | .506^***^ | .572^***^ | .411^***^ | .603^***^ | .754^***^ | .540^***^ | .501^***^ | .566^***^ | .582^***^ | .630^***^ | 1 |  |  |  |  |  |  |  |
| Q12 | .625^***^ | .694^***^ | .535^***^ | .710^***^ | .548^***^ | .834^***^ | .634^***^ | .655^***^ | .590^***^ | .724^***^ | .613^***^ | 1 |  |  |  |  |  |  |
| Q13 | .665^***^ | .640^***^ | .480^***^ | .681^***^ | .470^***^ | .751^***^ | .704^***^ | .590^***^ | .471^***^ | .678^***^ | .517^***^ | .719^***^ | 1 |  |  |  |  |  |
| Q14 | .573^***^ | .724^***^ | .379^***^ | .605^***^ | .451^***^ | .604^***^ | .532^***^ | .701^***^ | .409^***^ | .582^***^ | .476^***^ | .618^***^ | .616^***^ | 1 |  |  |  |  |
| Q15 | .606^***^ | .638^***^ | .594^***^ | .699^***^ | .555^***^ | .660^***^ | .607^***^ | .623^***^ | .717^***^ | .697^***^ | .559^***^ | .675^***^ | .647^***^ | .600^***^ | 1 |  |  |  |
| Q16 | .526^***^ | .585^***^ | .467^***^ | .648^***^ | .529^***^ | .586^***^ | .597^***^ | .593^***^ | .524^***^ | .669^***^ | .558^***^ | .601^***^ | .597^***^ | .522^***^ | .623^***^ | 1 |  |  |
| Q17 | .481^***^ | .575^***^ | .425^***^ | .597^***^ | .726^***^ | .509^***^ | .541^***^ | .574^***^ | .587^***^ | .628^***^ | .740^***^ | .552^***^ | .509^***^ | .496^***^ | .599^***^ | .581^***^ | 1 |  |
| Q18 | .654^***^ | .693^***^ | .482^***^ | .744^***^ | .584^***^ | .744^***^ | .713^***^ | .616^***^ | .520^***^ | .736^***^ | .625^***^ | .747^***^ | .737^***^ | .606^***^ | .656^***^ | .629^***^ | .647^***^ | 1 |

Note. *** p-value < 0.001

| **Table S6. Measurement Invariance of the PPCS Across Gender** | | | | | | | | |
| --- | --- | --- | --- | --- | --- | --- | --- | --- |
| **Model** | **χ² (df)** | **CFI** | **TLI** | **RMSEA (90% CI)** | **SRMR** | **ΔCFI** | **ΔRMSEA** | **Decision** |
| Configural Model | 665.650 (252)*** | 0.988 | 0.985 | 0.059 (0.053–0.064) | 0.038 | – | – | Supported |
| Metric Invariance | 861.590 (336)*** | 0.985 | 0.986 | 0.057 (0.052–0.062) | 0.035 | -0.003 | -0.002 | Supported |
| Scalar Invariance | 572.601 (336)*** | 0.991 | 0.992 | 0.054 (0.046–0.061) | 0.034 | +0.006 | -0.003 | Supported |
| ***Note*:** χ² = Chi-square test; df = degrees of freedom; ***= p-value tests the null hypothesis of perfect model fit < .001; CFI = Comparative Fit Index (≥ 0.95 indicates good fit); TLI = Tucker-Lewis Index (≥ 0.95 indicates good fit); RMSEA = Root Mean Square Error of Approximation (≤ 0.08 suggests acceptable fit, ≤ 0.06 indicates good fit), with 90% confidence intervals reported; SRMR = Standardized Root Mean Square Residual (≤ 0.08 suggests good fit); ΔCFI = change in CFI, where values < 0.01 indicate invariance; ΔRMSEA = change in RMSEA, where values < 0.015 support invariance. Configural invariance tests whether the factor structure is the same across groups. Metric invariance constrains factor loadings to be equal, and scalar invariance further constrains item intercepts to test whether latent mean comparisons are valid. All levels of measurement invariance were supported*.* | | | | | | | | |
